# Generation and application of immortalized sheep fetal fibroblast cell line

**DOI:** 10.1186/s12917-024-04054-3

**Published:** 2024-05-14

**Authors:** Guoyu Du, Cheng Zhang, Xiaoan Cao, Lingxia Li, Yong Zhang, Youjun Shang, Jinyan Wu

**Affiliations:** 1 State Key Laboratory for Animal Disease Control and Prevention, Lanzhou Veterinary Research Institute, Chinese Academy of Agricultural Sciences, Lanzhou, 730046 China; 2 College of Veterinary Medicine, Gansu Agricultural University, Lanzhou, 730046 China; 3 College of Agriculture and Animal Husbandry, Qinghai University, Xining, 810016 China

**Keywords:** Application, Sheep fetal fibroblasts, ORFV vaccine strain, SV40 large T antigen, Immortalized

## Abstract

**Background:**

Primary sheep fetal fibroblasts (SFFCs) have emerged as a valuable resource for investigating the molecular and pathogenic mechanisms of orf viruses (ORFV). However, their utilization is considerably restricted due to the exorbitant expenses associated with their isolation and culture, their abbreviated lifespan, and the laborious procedure.

**Results:**

In our investigation, the primary SFFCs were obtained and immortalized by introducing a lentiviral recombinant plasmid containing the large T antigen from simian virus 40 (SV40). The expression of fibronectin and vimentin proteins, activity of SV40 large T antigen, cell proliferation assays, and analysis of programmed cell death revealed that the immortalized large T antigen SFFCs (TSFFCs) maintained the same physiological characteristics and biological functions as the primary SFFCs. Moreover, TSFFCs demonstrated robust resistance to apoptosis, extended lifespan, and enhanced proliferative activity compared to primary SFFCs. Notably, the primary SFFCs did not undergo in vitro transformation or exhibit any indications of malignancy in nude mice. Furthermore, the immortalized TSFFCs displayed live ORFV vaccine susceptibility.

**Conclusions:**

Immortalized TSFFCs present valuable in vitro models for exploring the characteristics of ORFV using various techniques. This indicates their potential for secure utilization in future studies involving virus isolation, vaccine development, and drug screening.

**Supplementary Information:**

The online version contains supplementary material available at 10.1186/s12917-024-04054-3.

## Introduction

The orf virus (ORFV) belongs to the Parapoxvirus genus of the Poxviridae family and can induce contagious diseases in small ruminant species. Orf is a zoonotic disease worldwide, affecting individuals who directly or indirectly come into contact with afflicted animals, including animal handlers, farmers, and veterinarians [1–3]. Currently, vaccine immunity is the principal approach for the prevention and control of orf, while treatment is a secondary alternative. Although conventional orf live vaccines may not offer sustained immune protection, immunizing animals with an orf live virus is still regarded as one of the most effective strategies. This approach can potentially decrease the severity of ORFV outbreaks in herds [4]. Orf viruses multiply in numerous primary cells, including bovine testicular cells (BTC) [5], fetal lamb testis cells (OA3.Ts) [6], and goat fetal fibroblast cells (GFFCs) [7], as well as passage cell lines, including bovine kidney cells (MDBK) [5] and Vero cells. Although ORFV can develop cytopathic effects (CPE) after multiple blind passages of MDBK and Vero cells, the resulting virus titers are insufficient to meet the criteria for virus isolation and vaccine production [8]. In our laboratory, we used primary sheep testicular Sertoli cells (STSCs) and primary sheep fetal fibroblasts (SFFCs) to reproduce an attenuated ORFV vaccine. Our findings indicated that ORFV production using SFFCs was easily cultured, yielding high titers of proliferating ORFV with high efficiency. However, after 15 in vitro passages, primary SFFCs underwent senescence, complicating their isolation and culture due to labor-intensive, expensive, and complex cell populations, leading to inaccurate viral titration. Moreover, biosafety concerns regarding tissue procurement may induce a potential risk of Brucella infection. Therefore, establishing an immortalized cell line capable of producing ORFV could offer a cost-effective and safe approach to manufacturing live ORFV vaccines.

This study aimed to establish and characterize an immortalized TSFFC cell line through SV40 large T (SV40T) gene introduction and cell line evaluation. The immortalized cells were assessed for morphological features, cell proliferation capacity, SV40T gene expression, analysis of SFFCs marker proteins, apoptosis testing, and their potential use in orf virus live vaccine production. Immortalized sheep fetal fibroblasts could also be crucial for future toxicology studies, co-culture experiments, transfection studies, vaccine development, and drug testing.

## Materials and methods

### Animals, cell line, antibody, reagent and virus strains

BALB/c-nude mice (50 days old) weighing 19–24 g were purchased from the Animal Experiment Center of Lanzhou Veterinary Research Institute, Chinese Academy of Agricultural Sciences. The live strain HCE of the ovine contagious pustular dermatitis vaccine was acquired from Shandong HuaHong Biological Engineering Co., Ltd. The vaccine strain was generated and prepared from primary sheep testicular Sertoli cells, and the viral titer of the lyophilized vaccine was ≥ 10^5^ TCID_50_/_0.1 mL_. It is used clinically to prevent ORFV infection in sheep and goats. Mouse ORFV-positive serum was produced by immunizing BABL/c mice with ORFV-attenuated strain ORFV-SC1 [9] and confirmed through ORFV neutralization experiments, showing a neutralizing virus titer of 1:128. The State Key Laboratory for Animal Disease Control and Prevention at the Lanzhou Veterinary Research Institute, Chinese Academy of Agricultural Sciences (our laboratory), provided SFFCs, STSCs [10], 293T cells, and Vero cells. HeLa cells were purchased from the BeNa culture collection. The antibodies and reagents used in this manuscript are listed in Table 1.

**Table 1:** Antibody and reagent information

Name	Cat No.	Brand
Vimentin (VIM) rabbit antibody	10366-1-AP	Proteintech
Fibronectin 1(FN1) rabbit polyclonal antibody	15613-1-AP	Proteintech
Cytokeratin5 (CK5) rabbit polyclonal antibody	26411-1-AP	Proteintech
SV40 Large T Antigen rabbit mAb	PA5-112036	Invitrogen
Beta-Actin antibody	TA7018S	Abmart
Goat anti-rabbit IgG	AP132	Sigma-Aldrich
Goat anti-rabbit IgG secondary antibody (FITC)	65-6111	ThermoFisher Scientific
Goat anti-mouse IgG secondary antibody (FITC)	62-6511	ThermoFisher Scientific
FxCycleTM PI/RNase staining solution	F10797	ThermoFisher Scientific
Cell Counting Kit-8	K1018-5	Apexbio
CCCP	C6700	Solarbio
Blasticidin	Ant-bl-05	Invivogen
Orf Virus (ORFV) Probe qPCR Kit	YB7C35600y	X-Y Biotechnology
Annexin V FITC Apop Dtec Kit I	556,547	BD-Pharmingen
Colchicamide	A600322-0100	BBI

### Construction and transfection of plenti6.2/v5-dest-sv40t lentiviral expression system

The 8.6 kbp plasmid DNA pLenti6.2/V5-DEST lentiviral vector, containing the SV40T gene (2.1 kb) purified by Nanjing GenScript Biotechnology Co., Ltd., was used. PLenti6.2/V5-DEST-SV40T and pLenti6.2-GW/EmGFP lentiviral expression vectors containing green fluorescent protein (GFP) were individually packaged using 293T cells. This process followed specific methods as referenced in “generation and application of immortalized Sertoli cell line from sheep testis” [10]. The cell immortalization method utilized in this study is consistent with that described in a previous publication in the Journal of Virological Methods titled “generation and application of immortalized Sertoli cell line from sheep testis,” authored by the same researcher.

### Screening for blasticidin concentration

Primary sheep fetal fibroblasts were plated in 6-well plates at a cell passage concentration of up to 80%. Blasticidin was added at 0–7 µg/mL concentrations and diluted in a normal medium to determine the cell’s median lethal dose. Two replicate wells were set up at each concentration to observe and record cell growth status. The blasticidin medium was changed every two days for 14 days of continuous culture.

### Detection of SV40T lentiviral titers

The virus in 2.1 was centrifuged at 12,000 rpm and 4 ℃ for 10 min to remove cell debris. Then, sucrose (3 mL) at a 50% concentration was added to the bottom of the centrifuge tube. The supernatant was evenly divided into ultracentrifuge tubes, ensuring the weight difference between each tube was 0.01 g. The tubes were centrifuged at 30,000 rpm at 4 ℃ for 3 h. The collected precipitate was dissolved in 1/50 (pre-centrifugal volume) of PBS, dispensed, and stored at − 80 ℃. 293T cells were seeded into 96-well plates at a density of 1 × 10^4^ cells/well. Concentrated viruses containing the GFP were diluted 10-fold (10^− 1^–10^− 10^) and added to the cells for up to 36 h in the presence of 8 µg/mL polybrene. After 72 h, the supernatant was removed, and the cells were fixed with acetone-formaldehyde at − 20 ℃ for 20 min. Following fixation, the cells were washed three times with PBST, and fluorescence counts were determined using a fluorescence microscope.

### SV40T lentiviral infection of primary SFFCs

After plating 1 × 10^5^ cells in a 6-well plate and reaching a cell density of 70%, the lentivirus was diluted using DMEM/F-12 standard medium. The multiplicity of infection (MOI) for the diluted lentivirus was set at 70, followed by adding polybrene at a concentration of 8 µg/mL to the cell wells. The cells were then incubated in a CO_2_ incubator at 37 ℃ for 2 h to facilitate adsorption. Subsequently, the medium was replaced, and the cells were cultured for 72 h. The selection was performed using blasticidin medium at a concentration of 4.0 µg/mL, and the medium was replaced every three days for 12 days. Once the cells reached capacity, the blasticidin concentration was increased to 4.5 µg/mL, and the screening process was repeated for another 12 days. Finally, blasticidin was replaced with the normal culture medium.

### Detection of protein markers and sv40t antigen expression in the primary SFFCS and immortalized TSFFCS

The protein expression of two markers, fibronectin (FN1) and vimentin (VIM), was analyzed to identify fibroblasts, with the absence of the epithelial cell marker cytokeratin (CK) [11–13]. This study particularly utilized the FN1 [14] and CK5 [15] antibodies. Immunofluorescence staining was used to assess the protein expression of FN1, VIM, and CK5 in primary SFFCs and 48th passage immortalized TSFFCs. Additionally, the expression of SV40T antigen was examined in the 6th passage primary SFFCs, and the 5th and 48th passage immortalized TSFFCs. The methodology detailed in Sect. 2.4 involved using VIM rabbit polyclonal antibody, FN1 rabbit polyclonal antibody, CK5 rabbit polyclonal antibody, and SV40T antigen rabbit mAb (1:100) as primary antibodies. Goat anti-rabbit IgG-fluorescein isothiocyanate (FITC; 1:100) was applied as the secondary antibody. Fluorescence microscopy was used to capture images. Furthermore, the SV40T gene was detected using western blotting. This experiment was performed on passage 6 SFFCs and immortalized TSFFCs at passages 20 and 48. The 293 T cells were employed to serve as positive controls. The primary antibody used was SV40T gene mAb (1:500), and the secondary antibody used was goat anti-rabbit IgG (1:2000). Images were acquired using a high-resolution image-acquisition system.

### Cell passage assay, cell viability assay, and cell cycle assay

Continuous cell cultivation was performed using immortalized TSFFCs at a passage ratio of 1:4. Each cell passage was grown to a confluent monolayer. The growth conditions of the passaged cells are listed in Table 2. The cells were cryopreserved in liquid nitrogen every 10th passage.

Proliferation assays were conducted on primary SFFC in passages 8 and 15 and immortalized cells in passage 50. Growth curves were generated to assess cell proliferation. The aforementioned cells were plated in 96-well plates at a density of 5 × 10^4^ cells/well, followed by incubation at 37 ℃ and 5% CO_2_. At 24, 36, 48, 60, 72, 84, and 96 h after plating, 10 µL CCK8 was added to each well, incubated for 3 h in a CO_2_ incubator, and OD_450_ nm values were determined.

To analyze the cell cycle, we used primary SFFCs from passages 8 and 15 and immortalized TSFFCs from passage 50 in their growth state. Cell monolayers were confluent and treated with 0.25% trypsin, followed by centrifugation at 1200 rpm for 5 min. After removing the supernatant, the cells were washed three times with PBS. Subsequently, PBS (300 µL) was used to resuspend the cells, pre-cooled absolute ethanol (700 µL) was added dropwise, and the cells were incubated at 4 ℃ for 18 h. The cells were then washed and stained following the instructions included in the cell cycle detection kit. Finally, flow cytometric analysis was conducted using CytoFLEX (Beckman Coulter).

### Apoptosis assay

Apoptotic cells were analyzed using an apoptosis detection kit (BD, USA) following the manufacturer’s instructions. Primary SFFCs at passage 8 and TSFFCs at passage 50 were exposed to the CCCP apoptosis inducer at a final concentration of 100 µM for 20 min at 24 and 48 h, respectively, and a control group was established. Trypsinization was performed using 0.25% trypsin. Subsequently, the cells were centrifuged at 1200 rpm for 5 min and resuspended in 500 µL of Fluosannexin-v apoptosis detection kit solution. Flow cytometry (CytoFLEX, Beckman) was used to examine the cells treated with annexin-FITC and propidium iodide (PI). Cell apoptosis bar graph analysis was performed using GraphPad Prism V5.0, and the data were then analyzed using Statistical Package for the Social Sciences (SPSS) for one-way ANOVA (Analysis of Variance) testing. *P* < 0.01 (**) was considered significant, and *P* < 0.001 (***) was deemed highly significant.

### Examination of SV40T-SFFCs karyotypes

Karyotyping was performed on primary SFFCs passage 8 and immortalized TSFFCs passages 5 and 51 when the cell confluence reached approximately 60%. To induce cell arrest, colchicamide (n-deacetyl-n-methylcolchicine) was added to the cells at a concentration of 10 µg/mL. After incubating for one hour at 37 ℃, the cells were subjected to trypsin digestion at mid-term. Following trypsinization, the cells were washed with PBS and incubated in a hypotonic solution at 37 ℃ for 20 min to induce lysis of the plasma membrane. For fixation, cell monolayers were treated with a 3:1 methanol solution for 30 min. After exposing the cells to trypsin, Giemsa staining was performed to visualize the G band. Each sample was analyzed by counting at least 100 mitoses to determine the chromosome count. Furthermore, a minimum of three mitotic morphologies were assessed for each sample.

### Teratogenicity test

Nine BALB/c-nude mice were divided into three groups, each containing three BALB/c-nude mice. Immortalized TSFFCs were collected at passage 51, and their density was adjusted to 1 × 10^6^ cells/mL. These cells were transplanted subcutaneously onto the right thigh of BALB/c nude mice aged 50 days, which did not exhibit any thymic lesions specific to pathogenic (SPF) conditions and were regularly monitored in aseptic environments. The positive control group comprised HeLa cells, whereas the negative control group comprised 9th passage SFFCs. The BALB/c-nude mice were maintained under sterile conditions for one month, during which tumor growth was observed. Besides, samples of normal muscle and tumor tissues were obtained from the site of cell inoculation for HE staining. Finally, the morphological characteristics of the tissues were examined under an inverted microscope.

### Live ORFV vaccine strain was propagated using immortalized TSFFCS

The freeze-dried ORFV vaccine (ovine contagious pustular dermatitis vaccine, HCE strain) was dissolved in 2 mL of maintenance medium and passed through a 0.22 μm filter. Next, virus (100 µL) was added, along with maintenance medium (3 mL), to the cells simultaneously. The virus-infected cells were placed in a 5% CO_2_ incubator for 2 h and then added a maintenance medium to 10 mL. The ORFV vaccine strain was injected into primary SFFCs passage 5 (SFFCs-P5), TSFFCs passage 5 (TSFFCs-P5), TSFFCs passage 70 (TSFFCs-P70), sheep testicular Sertoli cells passage 3 (STSCs-P3), and Vero cells for five consecutive passages. Normal cells were maintained comparably to those of the control.

### 50% tissue culture infective dose (TCID_50_)

The ORFV vaccine HCE strain underwent four passages in five different cell lines (SFFCs-P10, TSFFCs-P15, TSFFCs-P80, STSCs-P3, and Vero) before inoculation into the same cell lines. After vaccination, viruses were collected at 12, 24, 48, 72, and 96 h and subjected to freeze-thaw cycles. Subsequently, cells from the aforementioned cell lines were seeded into 96-well plates at a density of 5 × 10^4^ cells/well and incubated at 37 ℃ with 5% CO_2_ for 36 h. The cells were then washed with a serum-free medium. The ORFV vaccine viruses harvested from different cells at different time points were serially diluted from 10^–1^–10^–9^ in serum-free medium in centrifuge tubes. The diluted virus (100 µL) was added to a 96-well culture plate, including a negative control. The cytopathic effect was monitored for one week, and the TCID_50_ was calculated using the Reed-Muench method.

### ORFV vaccine strain identified by indirect immunofluorescence in cells

Before collection, ORFV vaccine viruses that had undergone five continuous passages were cultured in different cell lines, including SFFCs-P8, TSFFCs-P17, TSFFCs-P82, TSTSCs-P5, and Vero for 72 h. The collected viruses underwent two rounds of freezing and thawing before being used to infect different cells at an MOI of 0.1. Cell samples were obtained 12, 24, 48, 72, and 96 h post-infection and fixed in a 1:1 acetone-formaldehyde fixative for specific indirect immunofluorescence analysis, with uninfected cells serving as negative controls. The specific operational procedures are explained in Sect. 2.4. ORFV-positive mouse serum (1:1000) was used as the primary antibody, whereas goat anti-mouse FITC (1:100) was used as the secondary antibody. After 5 min staining of 4′,6-diamidino‐2‐phenylindole (DAPI) at 37 ℃, images were acquired using an inverted fluorescence microscope.

### Real-time PCR (qPCR)

The ORFV genome was extracted using a DNA extraction kit. The ORFV probe qPCR kit from Shanghai YBio-tech was used to detect the DNA of the 0.1 MOI ORFV vaccine strain in infected SFFCs-P10, TSFFCs-P15, TSFFCs-P80, STSCs-P3, and Vero cells at 0, 12, 24, 48, 72, and 96 h. The PCR mixture comprised a 20 µL volume, with 10 µL 2× Probe qPCR MagicMix, 2 µL primer mixture, 1 µL probe, and 7 µL template. The amplification process followed the guidelines outlined in the instruction manual. All samples’ cycle threshold (Ct) values were converted into copy numbers according to the standard curve y = − 0.2998X + 13.038 *R* = 0.9995. Quantitative real-time PCR analysis was conducted using the ABI 7500 Real-Time PCR System (Applied Biosystems, Thermo Fisher Scientific, Waltham, MA, USA). Virus titration growth curves and viral copy number bar graph analysis were performed using GraphPad Prism V5.0. The data were analyzed using SPSS for one-way ANOVA testing. *P* < 0.01 (**) was considered significant, and *P* < 0.001 (***) was considered highly significant.

### TSFFCs passage and orfv replication assay

Immortalized TSFFC passage cells (documented in Sect. 2.7) were inoculated with the 5th passage ORFV/HCE strain virus at a multiplicity of infection of 0.1 MOI. The time taken for 90% CPE after each virus inoculation was recorded, along with the virus titers obtained from each passage of cells post-inoculation. The harvested viral titers of each generation of passaged cells after viral inoculation were determined. The differences in ORFV replication in various generations of passagedcells were compared.

## Results

### Immortalization of primary sheep fetal fibroblast cells

**Fig. 1:**
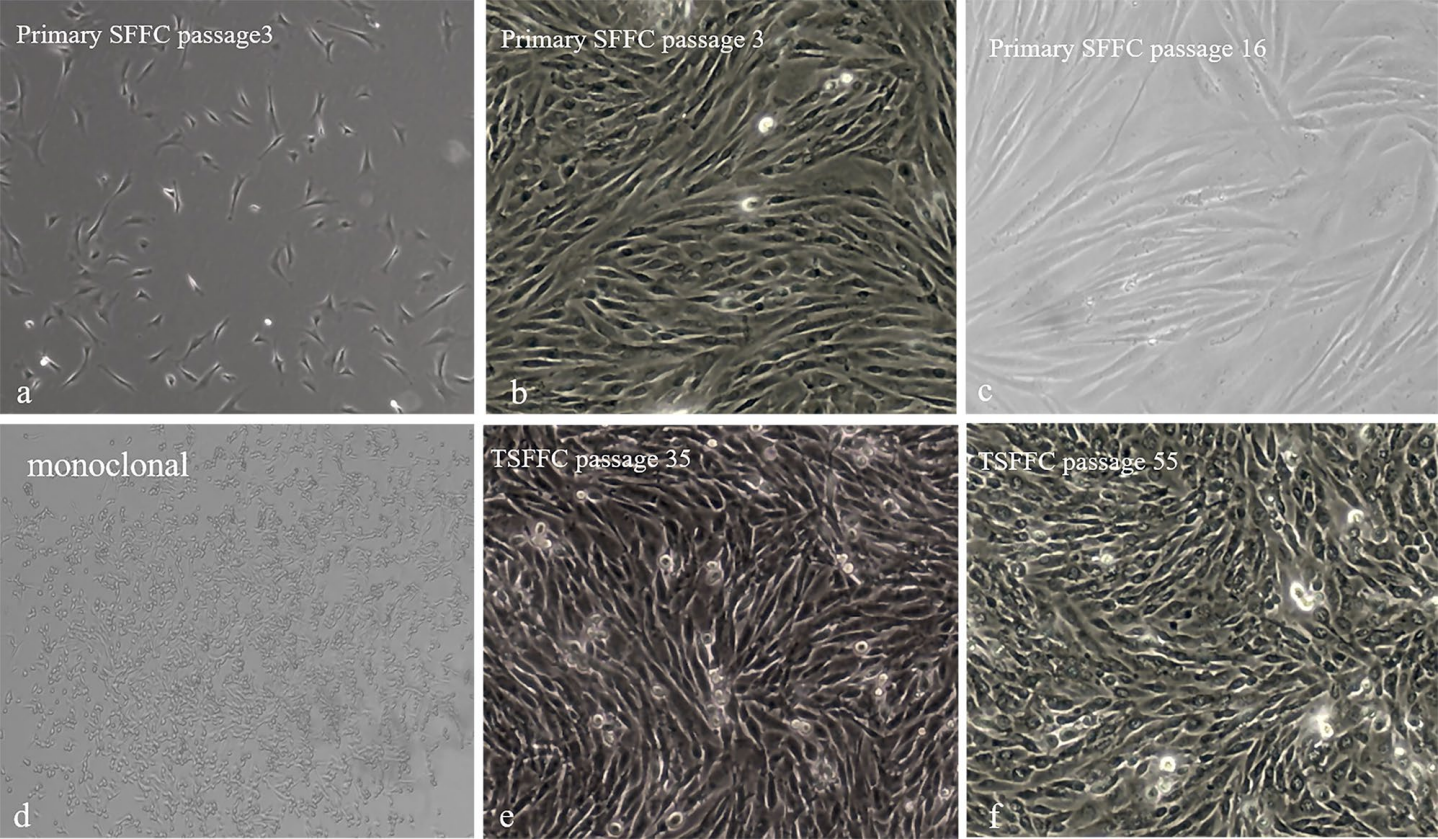
Morphological features of the primary SFFCs and immortalized TSFFCs. (**a**) SFFCs at passage 3 after 12 h of growth (100× magnification). (**b**) Morphology of primary SFFCs at passage 3 (100× magnification) after 36 h of culture. (**c**) Morphology of primary SFFCs at passage 16 (100× magnification). (**d**) Blasticidin-resistant cell clone (100× magnification). (**e**) Morphology of the TSFFCs at passage 35 (100× magnification). (**f**) Morphology of the TSFFCs at passage 55 (100× magnification)

**Fig. 2:**
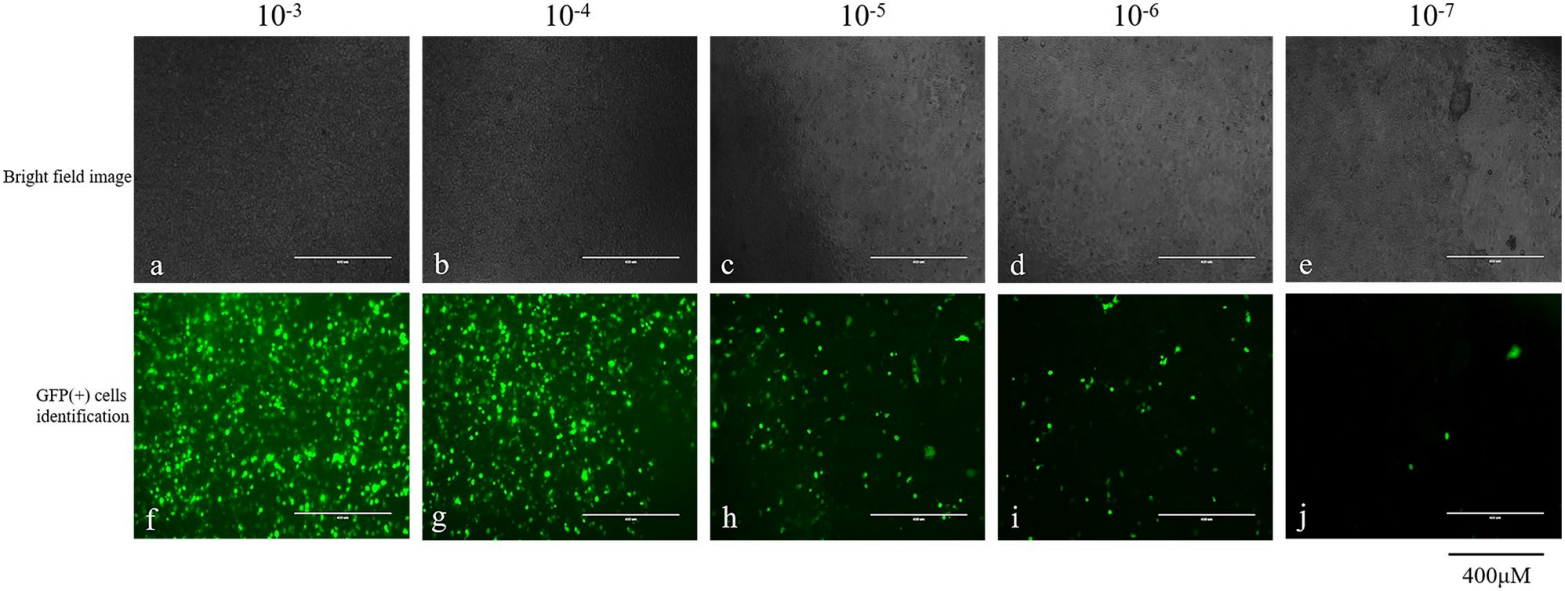
SV40T lentiviral titers. Microscopic images a, b, c, d, and e display dilutions (10^− 3^-10^− 7^) of SV40T virus packaged in 293T cells for cell infection. Images f, g, h, i, and j show the corresponding green fluorescence

The primary sheep fetal fibroblasts in this study presented distinct polygons and spindles (Fig. 1a). Early passage (1–10) cells covered the bottom of the culture flask at 36 h and were densely interconnected. The primary SFFC proliferation rate decreased after the 10th passage, and the intercellular distance increased at 48 h despite the cells maintaining a spindle shape and growing longer (Fig. 1b-c). Immortalized SFFC cell lines were established to prolong the primary cell lifespan and impede the rate of cellular senescence. The titer of the lentivirus packaged in this study was measured to be 3 × 10^7^ Tu/mL using the pLenti6.2-GW/EmGFP-positive plasmid (Fig. 2). The pLenti6.2/V5-DEST-SV40T plasmid MOI was adjusted to 70, and second-generation SFFC cells were transfected and treated with blasticidin for 12 days to obtain immortalized SFFCs. Monoclonal cell lines were generated when only a few cells transfected with the SV40T gene survived exposure to two concentrations of blasticidin (4.0 and 4.5 µg/mL; Fig. 1d). However, all existing control cells perished during the process. This has resulted in the establishment of a new immortalized cell line, TSFFCs. The proliferative ability of immortalized TSFFC cells was evaluated through continuous culture. Notably, the cell passage ratio of the immortalized TSFFCs was fixed at 1:5, and the cell growth time to confluent monolayer cells increased from around 36 h at the 10th passage to approximately 84 h at the 90th passage (Table 2). When compared, primary SFFC cells had a passage ratio of only 1:3, as increased ratios resulted in slower growth and hindered the expected fusion. Cell morphology variations were observed during passages, with TSFFC cells maintaining their typical polygonal and spindle shapes until the 35th passage (Fig. 1e). The cell morphology transformed at the 50th passage, with loss of spindle shape and cell shortening. However, the growth rate remained consistent, reaching confluence within 48 h (Fig. 1f). After approximately 100 generations, cell growth decelerated, cell status deteriorated, and the anticipated fusion was no longer accomplished.

### Validation of the immortalized TSFFCs

**Fig. 3:**
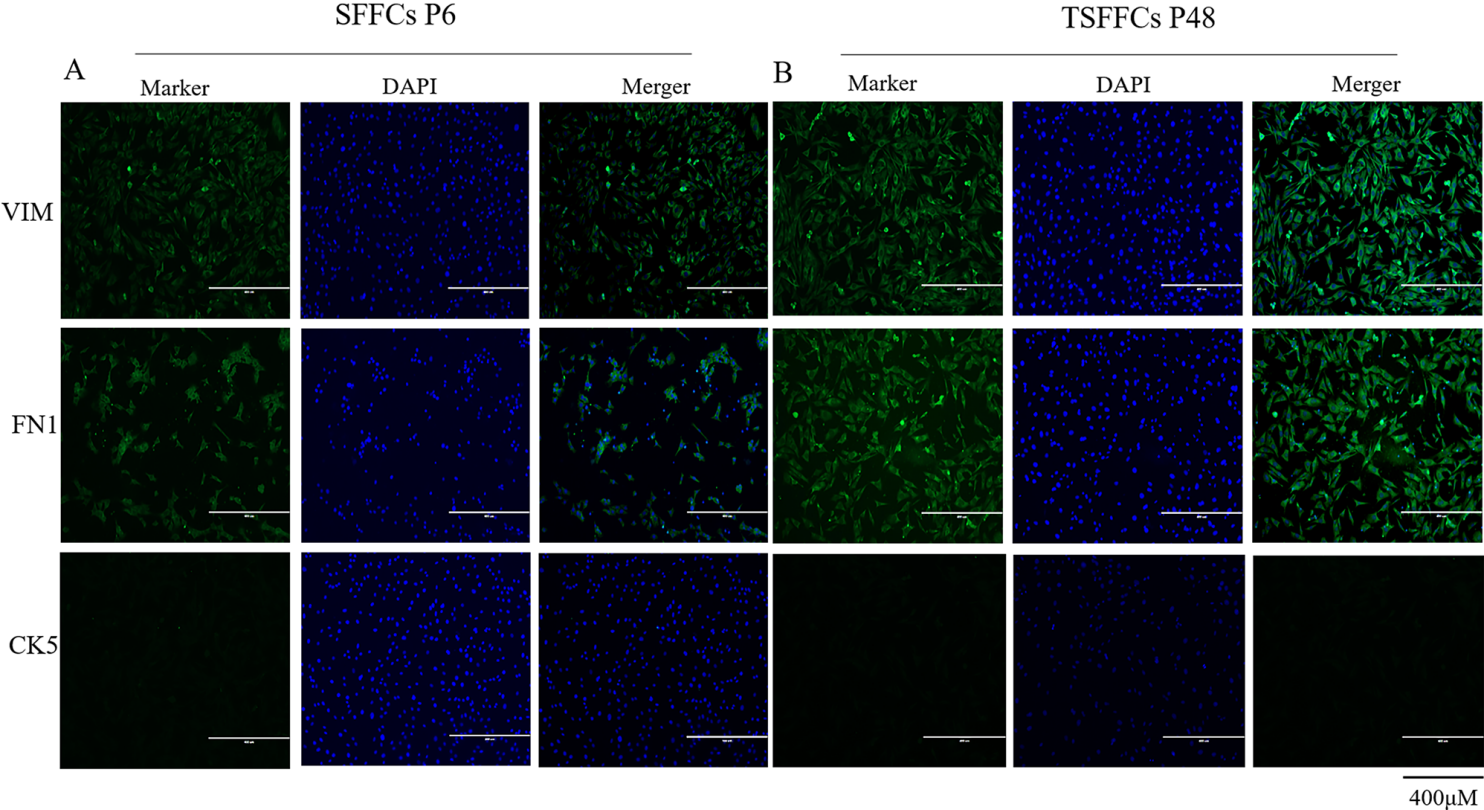
Identification of primary SFFCs and immortalized TSFFCs. (**A**-**B**) Immunofluorescence analysis of SFFCs at passage 6 and TSFFCs at passage 48 stained with FN1, VIM, and CK5 (200× magnification)

**Fig. 4:**
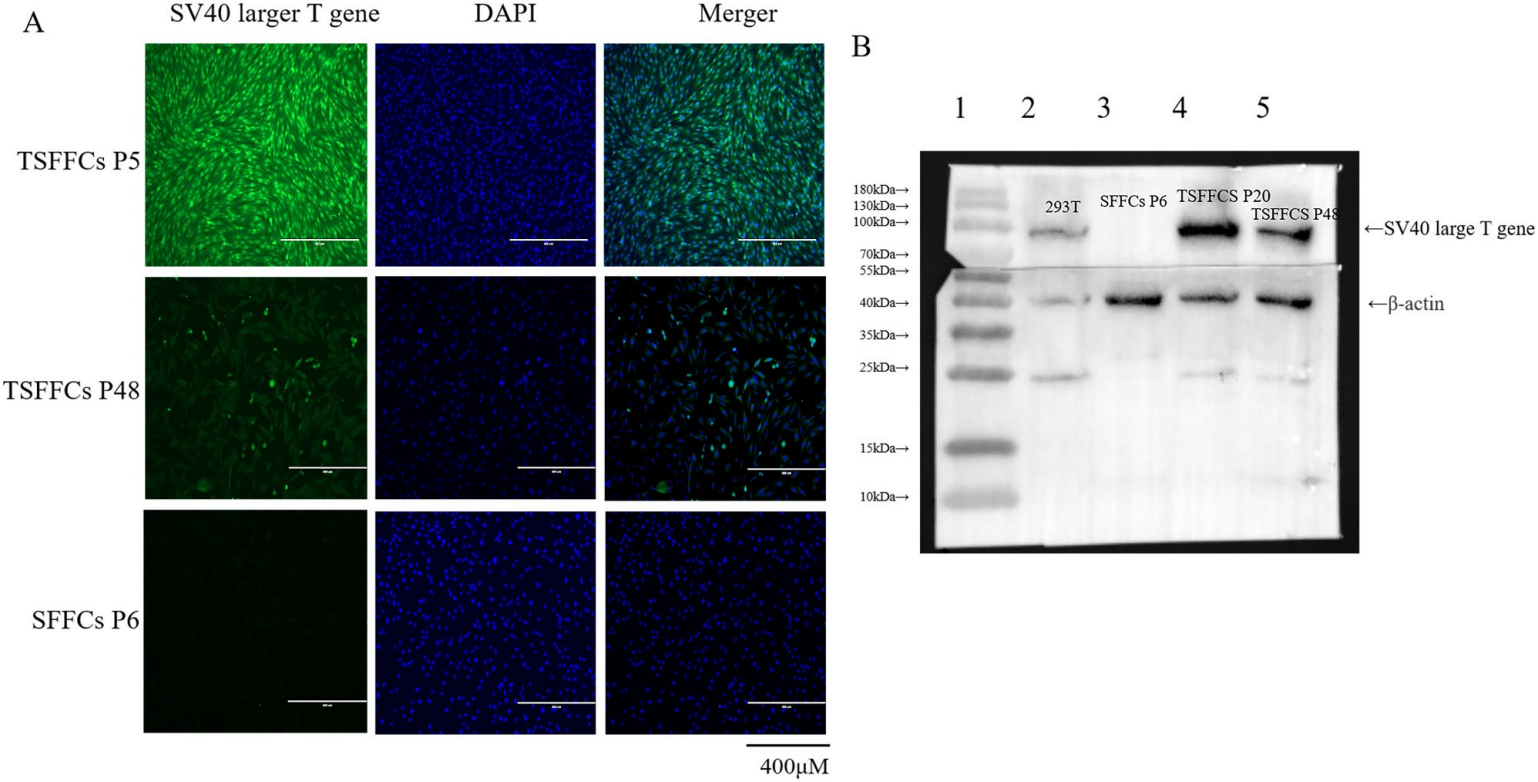
Identification of SV40T genes in TSFFCs. (**A**) Immunofluorescence identification. SFFCs at passage 6 and TSFFCs at passages 5 and 48 were stained with SV40T antigen (200× magnification). (**B**) Western blotting detection of the SV40T antigen in primary SFFCs and immortalized TSFFCs. Line 1: protein marker (purchased from Vazyme); line 2: 293T cells; line 3: SFFCs at passage 6; lines 4 and 5: TSFFCs at passages 20 and 48, respectively

Cytokeratin is an epithelial cell marker, whereas vimentin and fibronectin are expressed by fibroblasts. An immunofluorescence assay determined the expression levels of VIM, FN1, and CK5 proteins in primary SFFCs and immortalized TSFFCs at the 48th passage. Both VIM and FN1 proteins were observed in both cell types, indicating consistency in the characteristics and functions of immortalized TSFFCs with primary cells. Furthermore, the absence of green fluorescence in the CK5 protein indicated no contamination of epithelial cells in either SFFCs or TSFFCs (Fig. 3A-B). Immunoblotting and immunofluorescence assays detected the presence of SV40T antigen in immortalized TSFFCs. Immunofluorescence assays revealed significantly higher expression of SV40T antigen in the 5th -generation immortalized TSFFCs, while expression levels were noticeably reduced in 48th -generation TSFFCs (Fig. 4A). Correspondingly, western blotting analysis revealed the expression of SV40T antigen in the 20th and 48th generations of immortalized TSFFCs and 293T cells (positive control), with absence in the 6th generation of primary SFFCs (Fig. 4B). The expression of the SV40T antigen was relatively lower in the 48th generation than in the 20th generation. These results revealed that primary SFFCs do not naturally express SV40T antigen; the introduction of SV40T antigen causes cell immortalization, and with increasing passage numbers, the expression levels of SV40T antigen gradually decrease.

### Immortalized TSFFCs enhanced proliferation

**Fig. 5:**
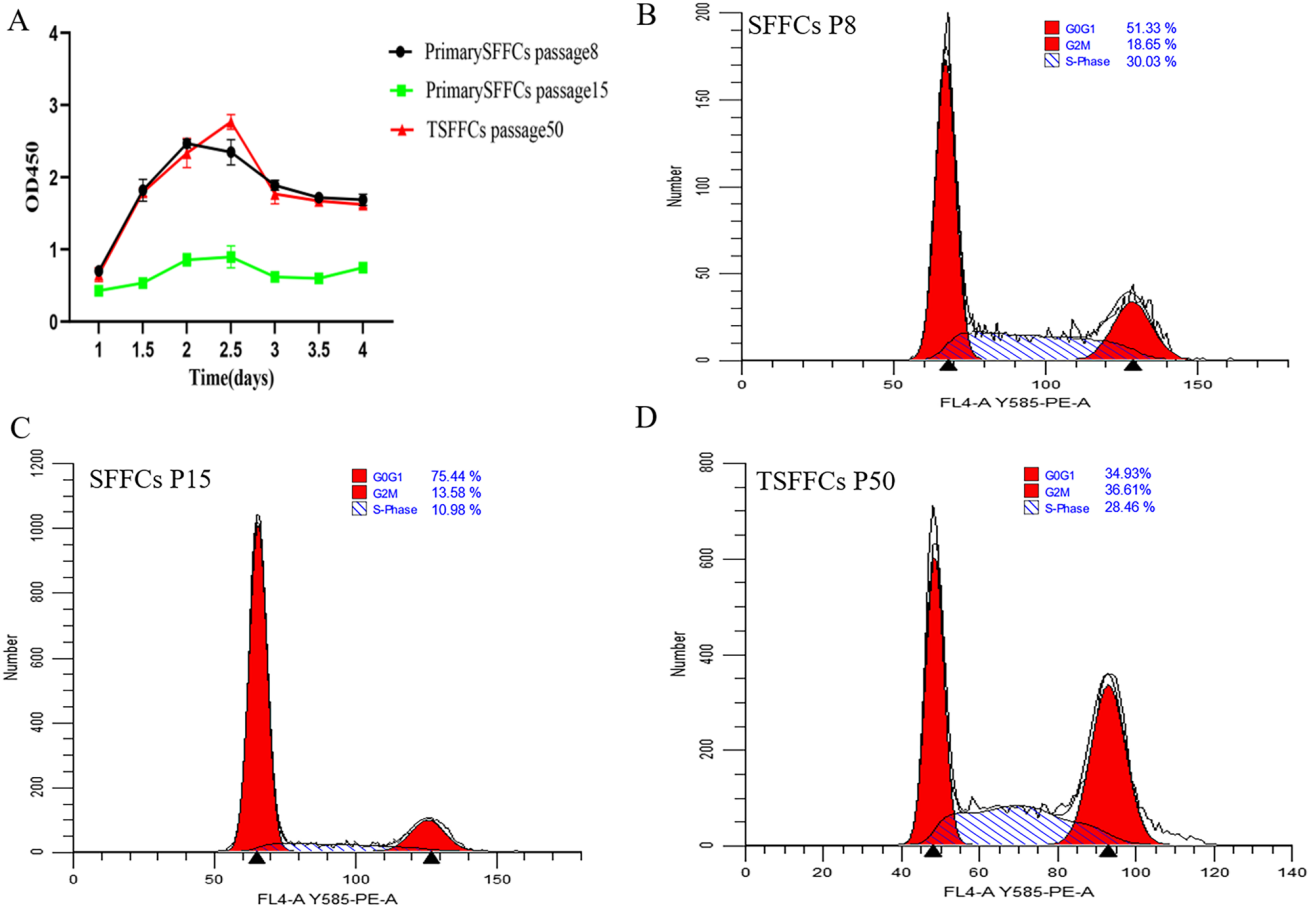
Growth curve and cell cycle analyses of primary SFFCs and immortalized TSFFCs. (**A**) Growth kinetics of SFFCs at passages 8 and 15, and TSFFCs at passage 50. (**B**–**D**) Cell cycle analysis of SFFCs at passages 8 and 15 and TSFFCs at passage 50

A growth curve and cell cycle analyses were performed to evaluate the proliferation potential of immortalized TSFFCs. The growth curve was detected using the CCK8 (Fig. 5A). The proliferation trend of immortalized TSFFCs at the 50th passage was comparable to that of the 8th passage SFFCs, exhibiting an S-shaped growth curve with a rapid cell proliferation increase after 36 h of passaging. At the 8th passage, SFFCs reached a peak proliferation rate 48 h post-passaging, while the immortalized TSFFCs at the 50th passage reached their maximum growth rate 2.5 days before declining. Alternatively, for the primary SFFCs in the 15th passage, there was minimal change in the proliferation rate within 1–4 days post-passaging. This rate significantly decreased compared to the 8th passage, indicating a gradual loss of differentiation ability of primary SFFCs after the 15th passage. To assess the impact of SV40T antigen expression on replicative lifetime and cell cycle progression, we examined the cell cycle distribution using flow cytometry. Differences in cycle phases were observed between the 8th and 15th passages of primary SFFCs (Fig. 5B-C) and between the 50th passage of immortalized TSFFCs (Fig. 5D). The proportion of cells in the S-phase in the 8th passage of primary SFFCs was 30.03%, which was 10.98% higher than that in the 15th passage. In the TSFFC after the 50th passage, the proportion of cells in the S-phase was 28.46%. These findings imply that the TSFFC cell line has a strong potential to proliferate and possesses a longer replicative lifespan than primary SFFCs up to 15 passages.

### Immortalized TSFFC resistant to apoptosis

**Fig. 6:**
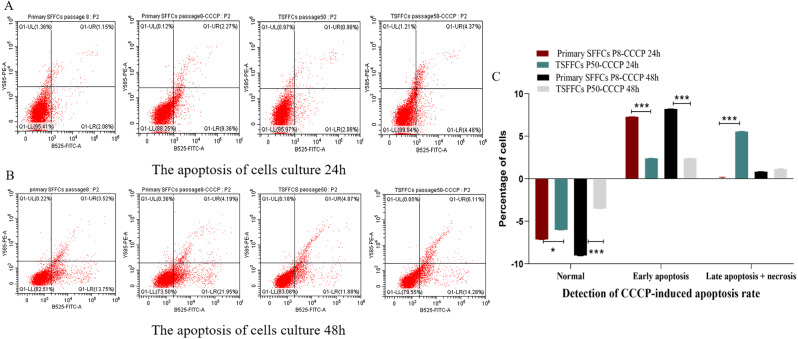
Apoptosis rates of primary SFFCs and immortalized TSFFCs induced by CCCP apoptosis inducers. (**A**) The apoptosis rate after 24 h. (**B**) Apoptosis at 48 h. (**C**) Differential analysis of apoptosis rate at 24 and 48 h between primary SFFCs and immortalized TSFFCs induced by the CCCP apoptosis inducer. Data are presented as mean ± SEM and analyzed using one-way ANOVA, *P* < 0.001(***), *P* < 0.01 (**)

Apoptosis, also known as programmed cell death, is a distinct form of cell death that plays a crucial role in maintaining the health of organisms [16]. To investigate the impact of cell immortalization on the rate of cell apoptosis, we measured the apoptosis rates of the two cell lines cultured for 24 and 48 h (Fig. 6A–C). Upon inducer addition, both cell types showed a reduction in the normal cell number at 24 and 48 h compared to the control group. SFFCs-P8 decreased by 7.16% and 9.01% at 24 and 48 h, respectively, whereas TSFFCs-P50 decreased by 6.03% and 3.53%, respectively. Early apoptosis detection results indicated that SFFCs-P8 exhibited a significantly higher early apoptosis rate after inducer addition than TSFFCs-P50 at 24 and 48 h (7.28%, 8.2%, 2.4%, and 2.4%). Furthermore, in the assessment of cell necrosis and late apoptosis rates, SFFCs-P8 showed minimal changes at 24 and 48 h after apoptosis induction, with decreases of − 0.12% and 0.81%, respectively, while TSFFCs-P50 demonstrated significant increases of 5.57% and 1.11%. Data on the degree of cell apoptosis indicated that immortalized TSFFCs exhibited a protective effect against early cell apoptosis compared with primary SFFCs. All the above data were obtained by subtracting the percentage of cells in the untreated group from the percentage of cells in the CCCP-induced group.

### Karyotype examinations and tumorigenicity assays

**Fig. 7:**
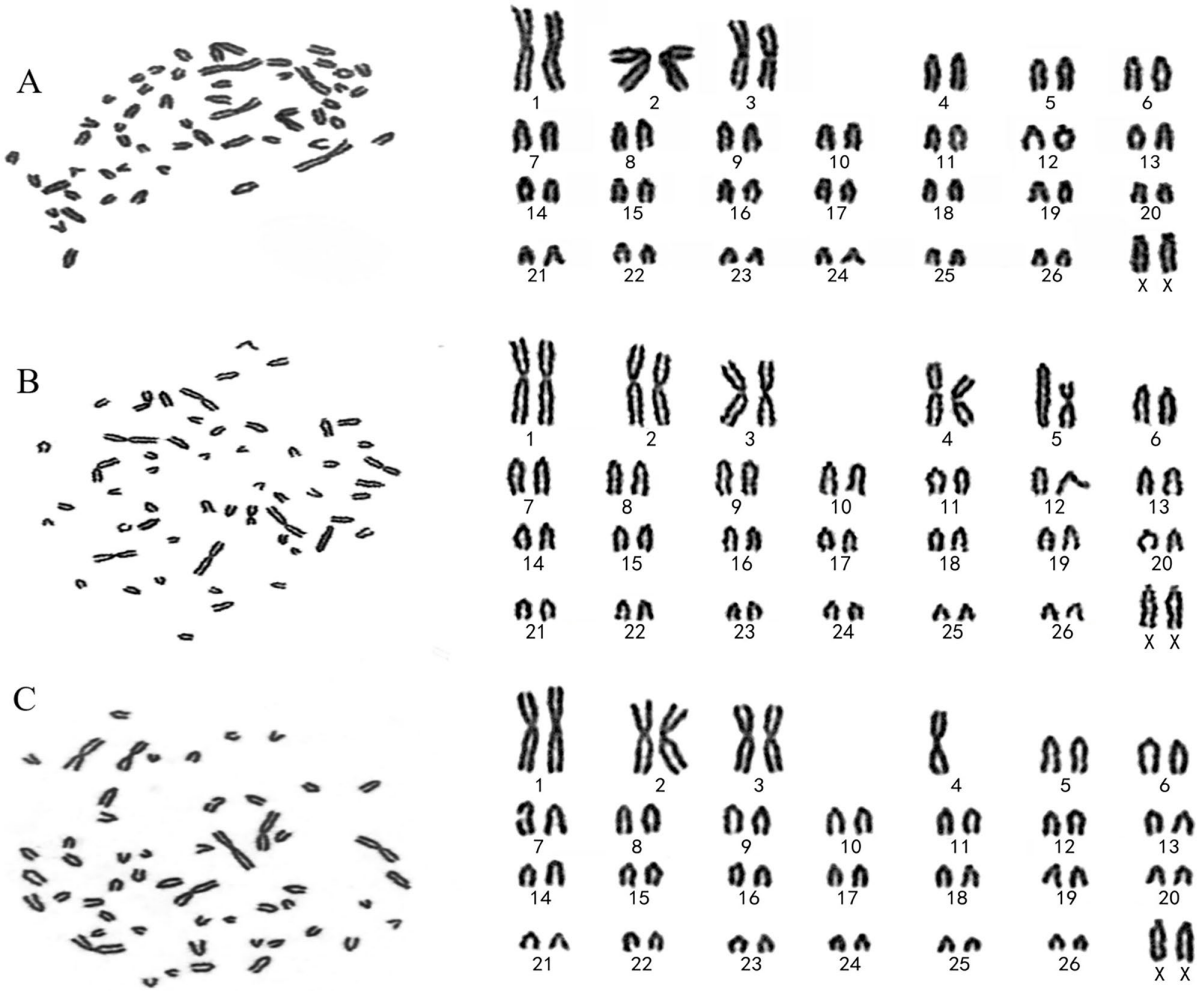
Karyotype analysis of primary SFFCs and immortalized TSFFCs. (A–C) Karyotype analysis of SFFCs at passage 8 and TSFFCs at passages 5 and 51. All data showed normal chromosome numbers (2n = 52 + XX patterns). However, telocentric chromosome centromere fusion has also been observed in TSFFCs

**Fig. 8:**
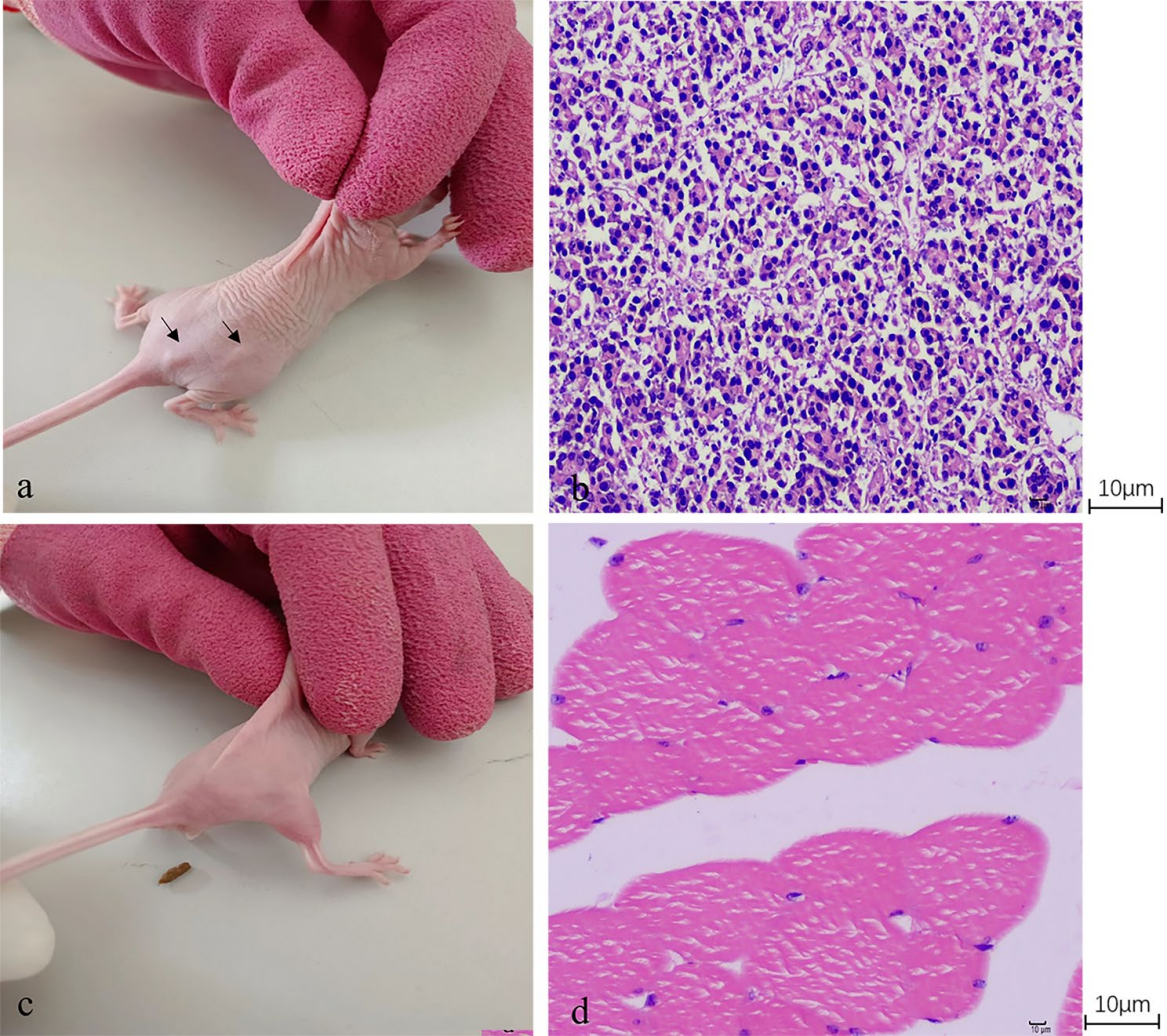
(**a**) BALB/c-nude mice injected with positive control HeLa cells developed tumors at the injection site on the 14th day. (**b**) Histological examination demonstrated a dense inflammatory cellular mass and high nuclear/cytoplasmic ratios below the HeLa cell injection site (400× magnification). (**c**) No tumors were detected in BALB/c-nude mice inoculated with TSFFCs at passage 51. (**d**) Normal tissue was observed at the injection site of TSFFCs at passage 51 (400× magnification)

To analyze the karyotypic stability of immortalized TSFFCSs, we selected actively growing primary SFFCs in passage 8 and immortalized TSFFCs in passages 5 and 51. Primary SFFCs and TSFFCs exhibited a standard and indicative karyotype, demonstrating the presence of a typical number of diploid chromosomes (2n = 54) in sheep (Fig. 7A–C). This included 26 pairs of autosomes and one pair of sex chromosomes corresponding to female sheep. Karyotype analysis revealed that both primary SFFCs and immortalized TSFFCs could maintain stable replication of chromosome numbers. The cells exhibited 54 chromosomes, and the structure and number of primary SFFCs remained consistent (Fig. 7A). The chromosome count in immortalized TSFFCs at passages 5 and 51 remained unchanged. However, abnormalities were observed in the chromosome structure of chromosomes 4 and 5 in the TSFFCs-P5 passage, including chromosome centromere fusion. In the TSFFCs-P51 generation, abnormalities were found in chromosomes 4 and 7, with chromosome 4 showing centromere fusion and chromosome 7 exhibiting chromatid breakage (Fig. 7B-C). To investigate the tumorigenic potential of the immortalized TSFFCs in vivo, we conducted a rigorous tumorigenicity assay in eight-week-old BALB/c-nude mice. Subcutaneous injection of TSFFCs at passage 51, primary SFFCs at passage 9 (serving as a negative control), and HeLa cells (as a positive control) were administered. After 14 days, tumors were observed in mice injected with HeLa cells (positive control; Fig. 8a). In sharp contrast, injection of primary SFFCs (data not shown) and TSFFCs (Fig. 8c) did not result in tumor formation. Histopathological examination of injection site samples validated these findings. Normal muscle tissue was observed at the site where primary SFFCs were injected (data not shown) and where TSFFCs were injected (Fig. 8d). Conversely, numerous densely infiltrating inflammatory cells and nucleated cytoplasmic cells were observed at the HeLa cell injection site (Fig. 8b). These results strongly support the conclusion that TSFFCs are non-tumorigenic and safe for further applications.

### Efficient replication of ORFV vaccine strain in TSFFCs

**Fig. 9:**
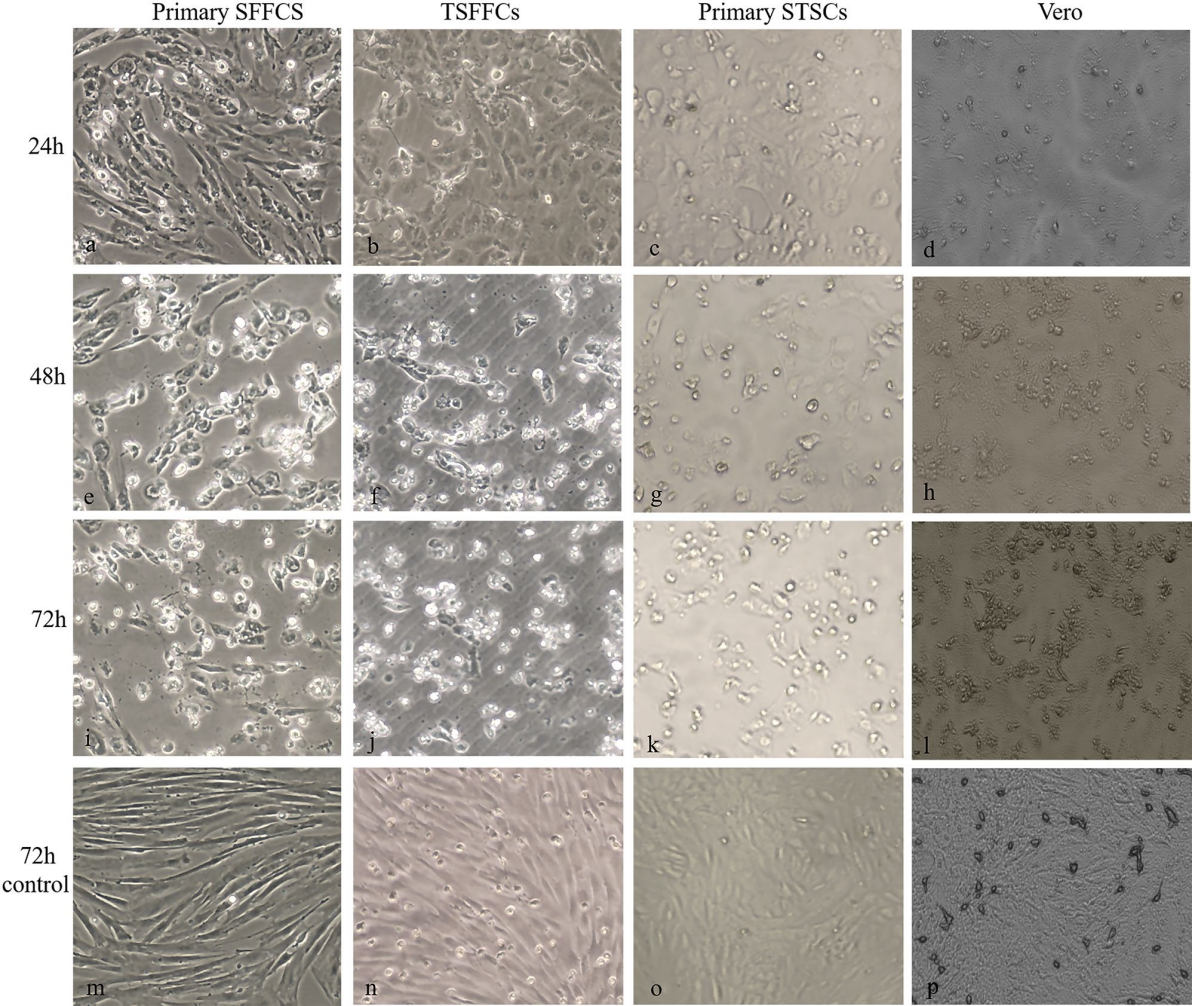
Microscopic observations of primary SFFCs, TSFFCs, primary STSCs, and Vero cells infected with ORFV vaccine HCE strains. (**a**–**d**) CPE of primary SFFCs, TSFFCs, primary STSCs, and Vero at 24 h. (**e**–**h**) CPE of primary SFFCs, TSFFCs, primary STSCs, and Vero at 48 h. (**i**–**l**) CPE of primary SFFCs, TSFFCs, primary STSCs, and Vero at 72 h. (**m**–**p**) Mock for ORFV vaccine strain-infected primary SFFCs, TSFFCs, primary STSCs, and Vero

**Fig. 10:**
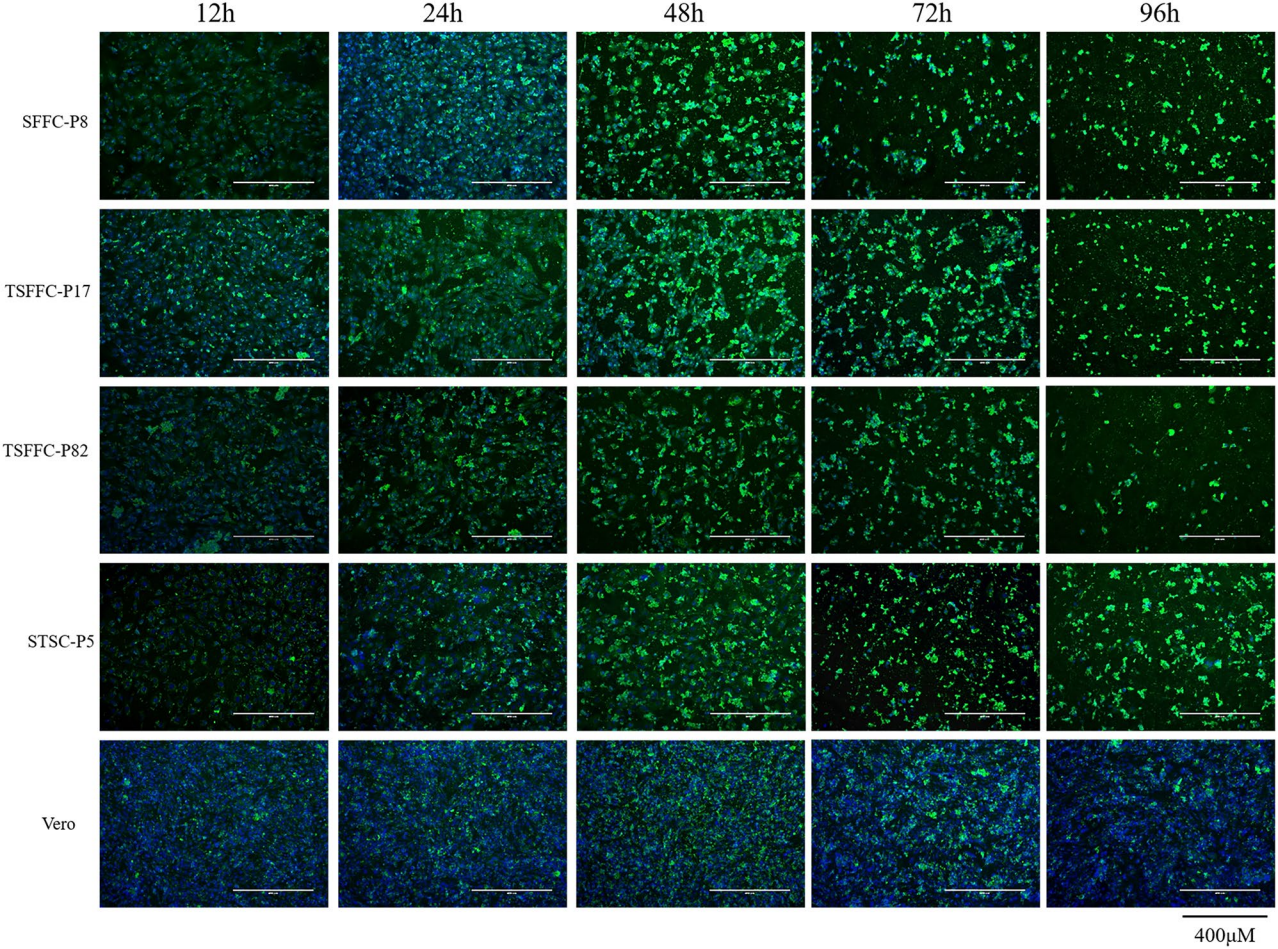
Immunofluorescence assays of primary SFFCs-P8, TSFFCs-P17, TSFFCs-P82, primary STSCs-P5, and Vero cells infected with ORFV vaccine HCE strain at 12, 24, 48, 72, and 96 h. Cells were stained with mouse ORFV-positive serum at 1:1000 dilution as a primary polyclonal antibody. Goat anti-mouse FITC antibodies were used as secondary antibodies (green). The nuclei were stained with DAPI (blue). All images were visualized and captured at 400× magnification DAPI: 4′,6-diamidino‐2‐phenylindole; FITC: fluorescein isothiocyanate

**Fig. 11:**
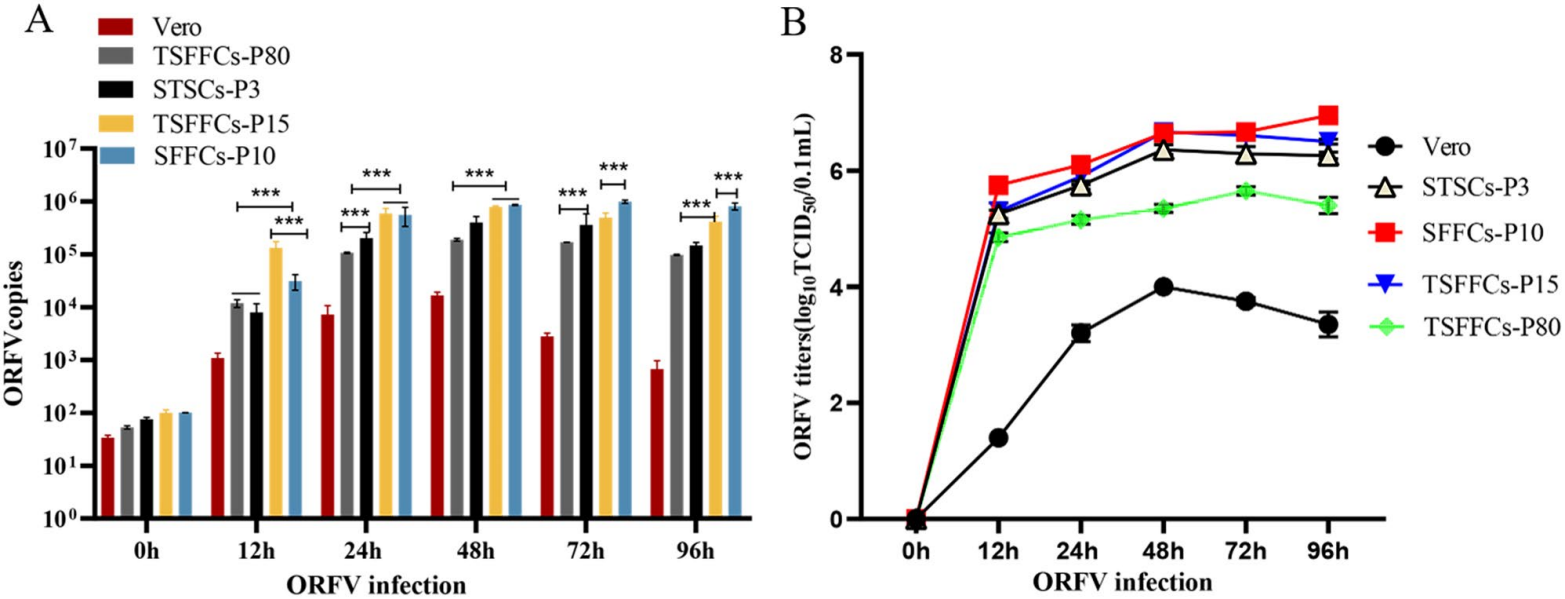
Infection with the ORFV vaccine HCE strain. (A) Real-time quantitative PCR analysis of ORFV vaccine strain replication in primary SFFCs-P10, TSFFCs-P15, TSFFCs-P80, primary STSCs-P3, and Vero cells at 12, 24, 48, 72, and 96 h. The data shown are representative of three independent experiments and are presented as mean ± SEM and analyzed using one-way ANOVA testing, *P* < 0.001(***), *P* < 0.01(**). (B) Illustration of virus titer curves for replicating ORFV vaccine HCE strains in primary SFFCs-P10, TSFFCs-P15, TSFFCs-P80, primary STSCs-P3, and Vero cells

Microscopic observation, indirect immunofluorescence assays, qPCR, and TCID_50_ were used to compare the propagation efficiency of ORFV vaccine strains in Vero, STSCs, SFFCs, and TSFFCs and to assess the sensitivity of these four cell types to the ORFV vaccine strain. Microscopic examination of these cell types infected with ORFV vaccine strains showed varying degrees of lesions at different times (Fig. 9). The intercellular space between the primary SFFCs and immortalized TSFFCs expanded, resulting in a modification in cell morphology 24 h after infection. Conversely, primary STSCs and Vero cells maintained their density and exhibited no obvious morphological changes. At 48 h post-infection, primary SFFCs and immortalized TSFFCs were entirely lysed, with only approximately 5% of normal cells surviving, whereas around 20% of primary STSCs and 40% of Vero cells retained their normal morphology. At 72 h post-infection, approximately 10% of normal cells remained in primary STSCs, whereas around 20% remained in Vero cells. Under consistent fluorescence intensity, various cell types, including TSFFCs-P17, TSFFCs-P82, primary STSCs, primary SFFCs, and Vero cells, exhibited distinct green fluorescence signals over time after infection with the ORFV vaccine HCE strain (Fig. 10). The green fluorescence intensity was strongest at 48 h after infection in primary SFFCs, TSFFCs-P17, TSFFCs-P82, and primary STSCs. At 72 h, cell integrity was compromised, reducing the cell nuclei’s blue fluorescence. At 96 h, primary SFFCs, and TSFFCs-P17 no longer exhibited nuclear staining, whereas TSFFCs-P82 and primary STSCs showed minimal blue nuclear staining. Furthermore, the fluorescence intensity of TSFFCs-P82 at different time points was consistently lower than that of the primary SFFCs, TSFFCs-P17, and primary STSCs. In Vero cells infected with the ORFV vaccine strain HCE, the green fluorescence gradually increased within 48 h, accompanied by a rise in the number of fluorescent cells. However, after 72 h, green fluorescence began to decline. The proliferation trends of the ORFV vaccine strain HCE in primary SFFCs, TSFFCs-P17, TSFFCs-P82, and primary STSCs were comparable. After 24 h, viral gene copies increased rapidly (Fig. 11A). Specifically, infection with the ORFV vaccine strain HCE in primary SFFCs and TSFFCs-P15 resulted in significantly higher viral gene copy numbers at 12, 24, 48, 72, and 96 h compared to infection in TSFFCs-P80 and primary STSCs. The viral copy number of the ORFV vaccine HCE strain inoculated in primary STSCs at 24, 48, and 72 h was higher than TSFFCs-P80. The peak viral gene copy value was observed 48 h post-infection in Vero cells. The viral genome copy number decreased as the infection progressed. Additionally, the TCID_50_ of viruses collected at various time points in different cells was calculated by assessing the CPE of the ORFV vaccine HCE strains on different cells. The standard growth curves of the HCE strains in various cells are illustrated in Fig. 11B. The TCID_50_ of the ORFV vaccine HCE strain increased in primary SFFC cells. The virus titer produced by primary SFFCs from 12 to 96 h was higher than other cells, reaching its peak at 96 h with a TCID_50_ value of 6.9 log10 TCID_50/0.1 mL_. The virus titer in primary STSCs and TSFFCs-P15 peaked at 48 h, with 6.34 log10 TCID_50/0.1 mL_ and 6.7 log10 TCID_50/0.1 mL_ values respectively. Interestingly, the TCID_50_ in TSFFCs-P15 cells surpassed that of primary STSCs from 12 to 96 h. When TSFFCs were passaged up to the 80th generation, the TCID_50_ of the ORFV HCE strain that was propagated decreased significantly, ultimately reaching a peak value of 5.32 log10 TCID_50/0.1 mL_ at 72 h. The viral titer of the ORFV vaccine HCE strain in Vero cells was significantly low. These findings indicate that the ORFV vaccine HCE strain replicates more effectively in early passage TSFFCs than in primary STSCs (the original HCE strain-replicating cell line), resulting in higher virus titers and infectious progeny virus production. The TCID_50_ results of the ORFV vaccine HCE strain propagated in TSFFCs of varying passages revealed that even when TSFFCs were passaged up to the 60th passage, the TCID_50_ (6.42 log10 TCID_50/0.1 mL_) of the virus produced remained higher than that of the primary STSCs (6.34 log10 TCID_50/0.1 mL_; Table 2).

**Table 2:** TSFFCs growth properties and virus replication

Passage number	Passage ratio	Cell growth time (to a confluent monolayer, d)	Time to 90% CPE(d)	Virus titers at 90% CPE (logTCID50/0.1mL)
10	1:5	1.5d	2d	6.67
20	1:5	1.5d	2d	6.73
30	1:5	1.5d	2d	6.70
40	1:5	1.5d	2d	6.65
50	1:5	2d	2d	6.58
60	1:5	2.5d	2d	6.42
70	1:5	3d	3d	5.87
80	1:5	3d	4d	5.32
90	1:5	3.5d	4d	4.91

## Discussion

The replication of orf viruses in various primary cells, including BTC, STC, GFFC, and OFTu, has been established. However, primary cells have a limited lifespan, tend to age, and die after a certain number of divisions [17]. This inherent instability, combined with the laborious and expensive isolation procedures associated with primary cells, contributes to the inconsistent quality of ORFV vaccines manufactured with them. Immortalized cell lines offer distinct advantages over primary cultures by maintaining consistent characteristics across multiple passages. This ensures uniformity in vaccine production as immortalized cells undergo repeated divisions and reach a stable physiological state [18]. Therefore, establishing cultured cell lines is crucial for developing ORFV vaccines. Various immortalization genes have been identified, with human telomerase reverse transcriptase (HTERT) and SV40T antigen being the most extensively studied. HTERT activates telomerase to prevent telomere shortening and achieve cell immortality [19], whereas SV40T antigen inhibits Rb through P53, preventing cell aging and apoptosis by promoting cell immortalization [20]. The SV40T antigen triggers the Rb protein inactivation (pRB, p130, and p107) and thereby activates E2F-dependent transcription, an increase in the S-phase ratio, cell proliferation, and, ultimately, immortality [21]. In practical applications, SV40 exhibits higher transfection and expression efficiency than HTERT [22]. Although the SV40T gene is crucial for the immortalization process, there are concerns regarding the potential loss of foreign genes during cell division [23]. To overcome this challenge, lentiviral vectors, a versatile gene transfer technique, have been employed to transport SV40T antigen. These vectors are safe and effective at transducing both dividing and non-dividing cells, facilitating the long-term expression of transgenes as the provirus genome is passed on to daughter cells. This long-lasting expression is vital for maintaining the transgene’s presence across multiple cell divisions [24, 25].

This study utilized packaged lentiviral vectors to introduce SV40T antigen into primary SFFCs for immortalization. Following blasticidin screening, immortalized cells exhibited enhanced growth rates and adhesion capabilities compared to conventional cells. Even after approximately 50 generations, TSFFCs retained the original characteristics of SFFCs, including the presence of characteristic proteins (VIM and FN1), whereas the absence of CK5 protein was consistent in both cell types. Analysis of SV40T gene expression in early and late passage TSFFCs using western blotting and indirect immunofluorescence revealed a gradual decrease in gene expression and weakening of green fluorescence as cells were passaged. This decline in gene expression is attributed to the random integration of foreign genes into the cellular genome by lentivirus [26], causing potential gene silencing through methylation in the CMV promoter region [27]. Notably, as methylation of the CMV promoter region increases over time during culture, foreign gene expression decreases [28]. Consequently, the time required for the TSFFC cells to grow into a dense monolayer gradually increases after the 50th passage. Even in the 50th generation of TSFFCs, despite decreased SV40T antigen expression, the proportion of S-phase cells in TSFFCs (28.46%) remained comparable to that in early passage cells of primary SFFCs (30.03%), indicating no significant difference. However, it was significantly higher than the S-phase proportion in late passage cells of primary SFFCs (10.98%). The early apoptosis rate induced by the apoptosis inducer CCCP in primary SFFCs was higher at 24 and 48 h than that in TSFFCs. As apoptosis culminates in cell death, late apoptotic cells exhibit damaged cell membranes [29], resulting in positive staining for both PI and FITC Annexin, rendering it difficult to distinguish between late apoptosis and necrotic cells. With no significant increase in late apoptosis and necrosis rates at 48 h, the notable increase at 24 h may be due to improper handling during cell digestion, causing membrane damage rather than an actual increase in late apoptosis rate. Consequently, it can be concluded that the introduction of the SV40T antigen confers TSFFCs with the ability to promote cell proliferation and inhibit apoptosis. Moreover, the SV40T gene can potentially induce chromosomal abnormalities and non-diploid cells, leading to increased genome instability and transformation. However, karyotype analysis in our study revealed that TSFFC cell lines maintained normal chromosome numbers despite abnormalities. Interestingly, no evidence of tumorigenic transformation was detected, suggesting the safe utilization of these cells in subsequent investigations.

ORFV can induce intracytoplasmic inclusions, disrupting cell monolayer continuity, forming multinucleated syncytia, and detaching cells with multiple nuclei [30]. This phenomenon can manifest in various cell types [5–8]. Recent research comparing the susceptibility of three different cell types to ORFV revealed that isolated newborn bovine testicular Sertoli cells were the most vulnerable to ORFV infection and exhibited the highest rate of viral replication. This study was limited to the initial 15 passages of these cells [5]. We compared the sensitivities of four different cell types to ORFV vaccine strains and assessed their potential for vaccine production. Our findings revealed that Vero cells exhibited delayed development of cytopathic effects compared to the other three cell types, along with lower viral titers and proliferation rates. Conversely, primary SFFCs exhibited heightened sensitivity to the ORFV vaccine strain, displaying complete cytopathic effects 48 h post-vaccination. Despite exhibiting the highest virus titer (6.9 log10 TCID_50/0.1 mL_) and proliferation rate, these cells were restricted to 15 generations of continuous culture. Immortalized TSFFCs, alternatively, exhibited the ability for indefinite passaging, albeit with a decrease in the proliferation rate with increasing passages. In TSFFC’s 60th generation, the virus titer generated by the ORFV HCE strain remained higher than that of the original STSCs. However, the viral titer and replication rate of the ORFV HCE strain decreased significantly at generations P70, P80, and P90. Although primary STSCs are frequently used in the commercial ORFV HCE vaccine production and yield high virus titers, they still fall short of the virus titers achieved by primary SFFCs and TSFFCs 60 generations ago. Primary STSCs could only be utilized up to P5. Therefore, the P10-P60 TSFFC cells are preferred for vaccine production and laboratory applications. Notably, TSFFCs utilize a more economical serum culture, resulting in a 33% lower production cost than that of SFFCs. With its superior virus propagation capacity and cost efficiency, immortalized TSFFC has emerged as a more favorable option for ORFV vaccine production.

In summary, an immortalized cell line, TSFFCs, derived from primary SFFCs, was effectively established by transfection with the lentiviral expression plasmid pLenti6.2/V5-DEST-SV40T. Compared to primary SFFCs, TSFFCs demonstrate consistent physiological properties, biological functions, and viral propagation capabilities, offering the advantages of virus production with consistent quality and stable titers. Implementing TSFFCs can streamline production and diagnostic processes, decrease production time and expense, and ensure the reproducibility of virus batches. Furthermore, it can minimize the risk of contamination by other pathogens during viral culture. These findings hold significant practical implications for industrial and large-scale vaccine production.

### Electronic supplementary material

Below is the link to the electronic supplementary material.

Supplementary Material 1

## Data Availability

The datasets used and/or analyzed during the current study are available from. the corresponding author on reasonable request.
