# Supplementary figures and images for: Generation and application of immortalized sheep fetal fibroblast cell line
Source: BMC Vet Res. 2024 May 14;20:198. doi: 10.1186/s12917-024-04054-3 (PMC11092253; doi:10.1186/s12917-024-04054-3)

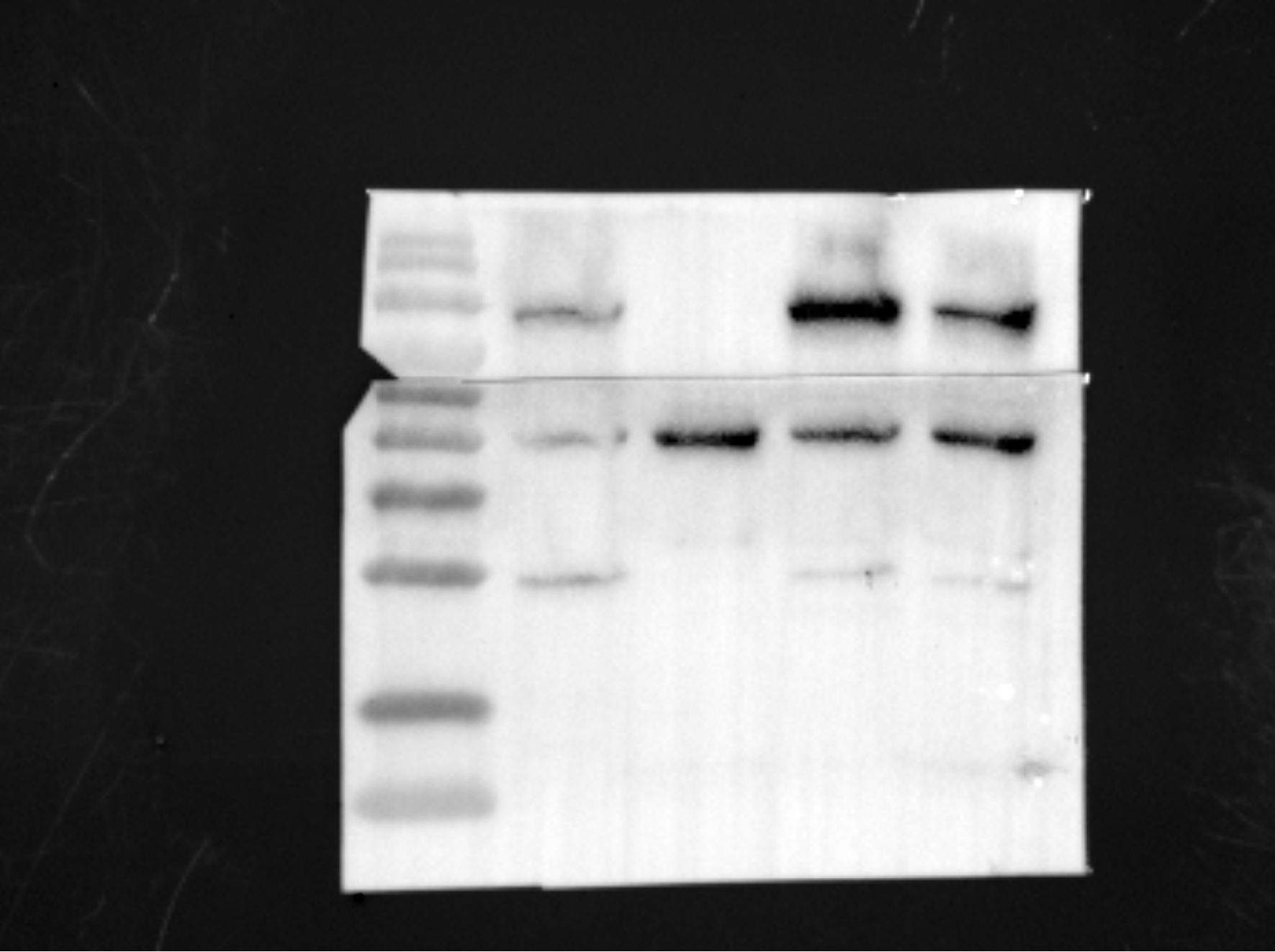

Supplement: Supplementary file 1 — Supplementary Material 1 [file 12917_2024_4054_MOESM1_ESM.tif]
